# Enhanced S-cone function with preserved rod function: a new clinical phenotype

**Published:** 2011-08-18

**Authors:** Michael Kinori, Eran Pras, Andrew Kolker, Gili Ferman-Attar, Iris Moroz, Joseph Moisseiev, Dikla Bandah-Rozenfeld, Liliana Mizrahi-Meissonnier, Dror Sharon, Ygal Rotenstreich

**Affiliations:** 1 Department of Ophthalmology, The Goldschleger Eye Institute, Sheba Medical Center, Tel Hashomer, Israel; 2 Sackler Faculty of Medicine, Tel Aviv University, Tel Aviv, Israel; 3 Department of Ophthalmology, Assaf Harofeh Medical Center, Zerifin, Israel; 4 Department of Ophthalmology, Hadassah—Hebrew University Medical Center, Jerusalem, Israel

## Abstract

**Purpose:**

To describe the clinical findings and genetic analysis in two brothers having a novel retinal disease characterized by an enhanced S-cone phenotype with normal rod function.

**Methods:**

Both patients underwent complete ophthalmologic examinations, including fundus photography, electroretinography (ERG), fluorescein angiography and optical coherence tomography (OCT). Mutation analysis of the following candidate genes was performed: nuclear receptor subfamily 2 group E member 3 (*NR2E3*), neural retina leucine zipper (*NRL*), nuclear receptor subfamily 1 group D member 1 (*NR1D1*), and thyroid hormone receptor beta (*THRB*).

**Results:**

Spectral photopic ERG responses demonstrated enhanced S-cone function in both patients. Their scotopic b-wave ERG amplitude responses, however, were within normal limits. Their scotopic a-wave amplitude responses were within the lower limit of normal. The a- and b-wave latencies were normal for one sibling and on the upper limit of normal for the other. Peripheral retinal findings were normal. OCT showed flattening of the macular curvature and thinning of the photoreceptor layer. Mutation analysis of *NR2E3*, *NRL*, *NR1D1,* and *THRB* genes was negative.

**Conclusions:**

We describe what appears to be a previously unidentified familial retinal phenotype with enhanced S-cone function and well preserved rod system function in contrast to the severely reduced rod function seen in the enhanced S-cone syndrome (ESCS). Genetic analysis of candidate genes did not reveal the cause of disease. We postulate that the disease might be caused by mutation of another, as yet unidentified gene, which encodes a protein that functions as a negative inhibitor of rod and S-cone development.

## Introduction

The sensitive short-wavelength cones (S-cones) comprise about 10% of the cone population in the adult human retina [1]. They differ from the sensitive long- and medium-wavelength cones (L- and M-cones, respectively) in function, anatomy, and retinal distribution [1,2]. Enhanced S-cone syndrome (ESCS) is a rare autosomal recessive disorder characterized by severely reduced function of rods, L cones and M cones, with enhanced S-cone function. Other characteristics are night blindness, cystoid maculopathy, and degenerative changes of the vascular arcades [3,4].

Full-field spectral (white and chromatic) electroretinogram (ERG) findings have shown that while patients with ESCS have severely reduced L- and M- cone responses, they show hypersensitive responses of the S-cones [5,6]. In addition, scotopic responses are severely diminished as a consequence of rod system involvement as well. Single-flash photopic responses, mediated by S-cones only, are within normal limits. The retinas of patients with ESCS are occupied by a larger than normal population of S-cones, which replace many of the rods and the L- and M-cones [7]. These S-cones are responsible for the normal or near-normal single-flash photopic ERG responses. Mutations in the photoreceptor cell-specific nuclear receptor subfamily 2 group E member 3 gene *NR2E3*, which is mapped to chromosome 15q24 and plays a role in photoreceptor differentiation and development, are found in a large majority of patients with ESCS [8].

We report a novel retinal phenotype in two adult siblings within an Arab family showing a typical ESCS photopic response on ERG but relatively normal scotopic ERG responses and normal retinal peripheral appearance. To the best of our knowledge, this particular set of symptoms has not been previously reported.

## Methods

### Patients

Two of ten adult siblings from an Arab family with consanguineous parents (first-order cousins) were studied. Both patients reported having reduced central vision, impaired color vision, and photophobia since childhood, but did not complain of impaired night vision. Patient 1 (P1) was a 45-year-old male who came to our outpatient clinic (Sheba Medical Center) complaining that over the past 5 years his vision had deteriorated. On admission to the clinic his best corrected visual acuity (BCVA) in both eyes was 1/20 with correction of −5.0 diopters. His 41-year-old brother, patient 2 (P2), also came to our clinic, complaining that his vision had deteriorated over the past 7 years. His BCVA on admission was 20/180 in the right eye and 20/600 in the left eye with correction of −7.0 diopters in both eyes. The medical histories of both brothers were otherwise unremarkable.

Both patients participated in the study after signing their informed consent to a protocol that was approved by the Institutional Review Board (Sheba Medical Center, Tel Hashomer, Israel). This study was approved by the review board of the Sheba Medical Center. This study conformed to the tenets of the Declaration of Helsinki and ARVO’s statements on human patients.

### Clinical studies

Ophthalmological examinations included BCVA, slit-lamp and dilated fundus examination, fundus photography, ERG, fluorescein angiography, and optical coherence tomography (OCT). The patients had been referred by their general eye practitioner in Umm al-Fahm, Israel.

Full-field ERG scans were obtained from both eyes of each patient by following the protocol of the International Society for Clinical Electrophysiology of Vision (ISCEV**)** [9]. After application of tropicamide 1% and phenylephrine 10% drops, the diameter of the dilated pupil was measured and the patient was dark-adapted for 30 min. A bipolar Burian-Allen electrode contact lens was placed in each eye. Scotopic responses were recorded for four single flash stimulus increment light intensities (0.023 cd-s/m^2^, 2.44 cd-s/m^2^, 23.5 cd-s/m^2^, and 252 cd-s/m^2^). For testing of photopic responses, the patient was then light-adapted for 10 min in white background light (29.63 cd/m^2^) before being exposed to a single white flash stimulus (2.44 cd-s/m^2^) and white 30-Hz flicker (2.44 cd-s/m^2^). Light-adapted spectral ERGs were recorded using a handheld full-field stimulator (Color Mini-Ganzfeld Stimulator [Kurbisfeld], LKC Technologies, Gaithersburg, MD) powered by colored light-emitting devices. Responses were evoked by red (627 nm), green (530 nm), and blue (470 nm) stimuli.

Stratus OCT 3 (Humphrey Instruments, Zeiss, Dublin, CA; version 4.0.1) scans were performed after pupils were dilated using macular thickness protocols and line scans at 0°, 25°, 90° and 125° at 6-mm and 9-mm lengths.

### Molecular genetic analyses

Genomic DNA was obtained from peripheral blood lymphocytes using the FlexiGene DNA kit (Qiagen). The direct sequencing method was applied for screening of coding exons as well as of flanking intron sequences of the *NR2E3,* neural retina leucine zipper *NRL,* thyroid hormone receptor beta *THRB,* and nuclear receptor subfamily 1 group D member 1 *NR1D1* genes. Amplification by PCR was conducted as previously described [10]. Primer sequences used to amplify the genomic fragments are listed in Table 1.

**Table 1:** Primers used to amplify candidate genes for the new retinal phenotype demonstrating enhanced S-cone function with preserved rod function: the nuclear receptor subfamily 2 group E member 3 (*NR2E3*), neural retina leucine zipper *(NRL),* thyroid hormone receptor beta *(THRB)* and nuclear receptor subfamily 1 group D member 1 (*NR1D1)* genes.

**Primer name**	**Gene**	**Exon**	**Sequence 5′-3′**	**PCR product size**
NR2E3–1F	*NR2E3*	1	CAGGCCTCCCGCAGGCAGGCAG	242
NR2E3–1R	*NR2E3*	1	CCATGGTCCCTGCGAACCTC	
NR2E3–2F	*NR2E3*	2	GAGGGGAGCGTGCAGCCCTG	248
NR2E3–2R	*NR2E3*	2	CACCCCTCCAGAACCCCTCAG	
NR2E3–3F	*NR2E3*	3	GTCCAAGCCCATGGCTCAGG	242
NR2E3–3R	*NR2E3*	3	GAAGGGTCAGGACGACACGC	
NR2E3–4F	*NR2E3*	4	GGGAGGTGACAAGAAATGGGC	327
NR2E3–4R	*NR2E3*	4	GAAGCCAAGCCCTGCTGTGC	
NR2E3–5F	*NR2E3*	5	CAAGTACTCCCTGCCACCTC	316
NR2E3–5R	*NR2E3*	5	GTGCCCTGTCTGGTTGACTC	
NR2E3–6F	*NR2E3*	6	GCTGTGTGTCTGCCATAACA	316
NR2E3–6R	*NR2E3*	6	CTCAAGGTTTGGGCAGAGAC	
NR2E3–7F	*NR2E3*	7	CTGTGCTAAGCTCACTGGTG	183
NR2E3–7R	*NR2E3*	7	GAGGTCAGGGACAGATGAGTG	
NR2E3–8F	*NR2E3*	8	GTCGTAAAACTGATGGCGTCCTC	232
NR2E3–8R	*NR2E3*	8	GCAAATGTTTCGTTTCAGTAGATTG	
NRL-1F	*NRL*	1	CACAGATGACCTCAGAGAGCTGGCCCTTTA	237
NRL-1R	*NRL*	1	CAGGTGTTAAAGAGGGGGTTCTAGGTGAGC	
NRL-2F	*NRL*	2	ACCATCCCTCTGGCTTTCCAAACTCTTGCT	683
MRL-2R	*NRL*	2	GATCTGATTGCTTTCAAGGGACCTTCTCCC	
NRL-3F	*NRL*	3	GACCTGGCGCTGACCCGGTTTCTGCATTCT	429
NRL-3R	*NRL*	3	GCCACCCCCACCAGCCCCCACTACACCACA	
THRB-1F	*THRB*	1	TGGAGAATGCATGCGTAGAC	439
THRB-1R	*THRB*	1	CGAAAACAAACAGTGAAACTTTG	
THRB-4F	*THRB*	4	AATGCATATGATATTGTTTGGAAC	264
THRB-4R	*THRB*	4	GGTTTGGAAATAACGGTTGC	
THRB-5F	*THRB*	5	CTTGCCTTCCAAAACTCTGC	261
THRB-5R	*THRB*	5	CACCATACATTGGAAGAGAAATG	
THRB-6F	*THRB*	6	TCTGGCCTAGCAACCTTAGC	101
THRB-6R	*THRB*	6	ACTGGGAGGGGACTGGAG	
THRB-7F	*THRB*	7	AGGTCCAAAACGATTCATCTC	148
THRB-7R	*THRB*	7	CCCAGTCGATCTCCTTGAAC	
THRB-8F	*THRB*	8	TTGCTGTGTATCTTGGGAGC	206
THRB-8R	*THRB*	8	TCCCAAGGTGATGAGGACTG	
THRB-9F	*THRB*	9	TTCAGAAGAGATTTTCTGCCAC	147
THRB-9R	*THRB*	9	TCGTTTTGTACTGACGTTGC	
THRB-10F	*THRB*	10	CAAATGTTAATCACAGAAGGTTATTCC	259
THRB-10R	*THRB*	10	AGCGCTAGACAAGCAAAAGC	
THRB-11F	*THRB*	11	ATTGGACAAAGCAAGCCTTC	242
THRB-11R	*THRB*	11	TGGAATGAAATGACACCCAG	
NR1D1–1F	*NR1D1*	1	TCTCTCTGCTCTTCCCATGC	361
NR1D1–1R	*NR1D1*	1	CACCCCAGTCCCTTACAAAG	
NR1D1–2F	*NR1D1*	2	GCTCCACATGGTGAACTGAG	553
NR1D1–2R	*NR1D1*	2	GTCCTGGCAAGACTGGTGTC	
NR1D1–3–4F	*NR1D1*	40606	CTTTTCCCTCCCTGGATCTC	532
NR1D1–3–4R	*NR1D1*	40606	CAGTATGATGTGTCTCCATTTGTG	
NR1D1–5F	*NR1D1*	5	GTGAAACCCCAAGCCTTCAG	850
NR1D1–5F	*NR1D1*	5	CACACTCAGCCTCCAGGAAC	
NR1D1–6F	*NR1D1*	6	CATCCTTCAGGGCCACAG	390
NR1D1–6F	*NR1D1*	6	AGATCGCACCATTGCACTC	
NR1D1–7F	*NR1D1*	7	TTCAGAAACAACCCCCACTC	345
NR1D1–7F	*NR1D1*	7	CCTACCTGCAGAGACAAGCAC	
NR1D1–8F	*NR1D1*	8	CAGGGAGAGGCTGCATTG	392
NR1D1–8R	*NR1D1*	8	TGGTTTGCTTTTCCTTTTCG	

Primers used to amplify candidate genes for the new retinal phenotype with enhanced S-cone function with preserved rod function: the *NR2E3,* neural retina leucine zipper *(NRL),* thyroid hormone receptor beta *(THRB)* and nuclear receptor subfamily 1 group D member 1 (*NR1D1)* genes.

## Results

**Figure 1:**
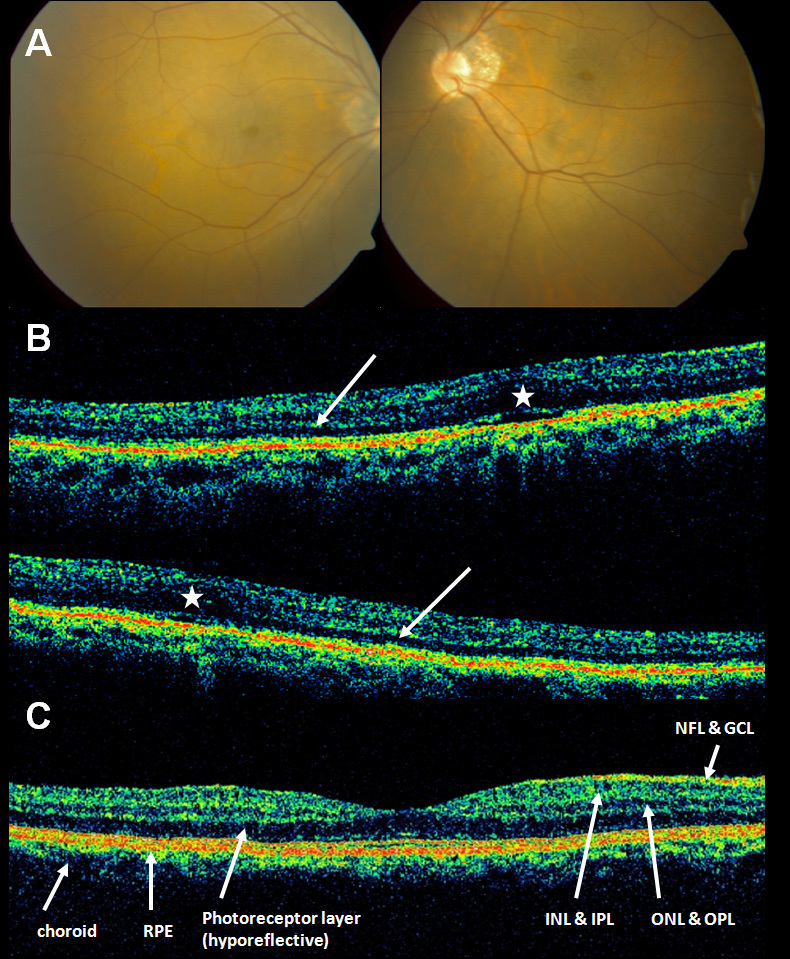
**A**: Fundus photography of patient 1 (P1) demonstrating bilateral cystoid-like macular lesions. There are no pigmentary changes. **B**: Optical coherence tomography (OCT) scans through the macular region of P1 (upper part) and P2 (lower part). There is loss of foveal contour (asterisks) and thinning of the hyporeflective layer representing the photoreceptors in the macular region (white arrows). P1 and P2 scans are off-center, but foveas can be seen. **C**: Reference areas within an OCT through the macular region of a normal patient. GCL, Ganglion cell layer; INL, inner nuclear layer; IPL, inner plexiform layer; NFL, nerve fiber layer; OPL, outer plexiform layer; RPE, retinal pigment epithelium.

In both patients, ophthalmic slit-lamp biomicroscopy revealed no lens opacity or degenerative vitreous changes in either eye. Fundus examination in both brothers demonstrated bilateral cystoid-like macular lesions with no pigmentary changes (Figure 1A demonstrates fundus findings in P1**)**. Fluorescein angiography showed no leakage in the macula in either of the brothers.

OCT of the macular area showed loss both of normal neuroretinal contours and of normal neuroretinal architecture. The photoreceptor layer showed severe thinning in the macular area (Figure 1B).

**Figure 2:**
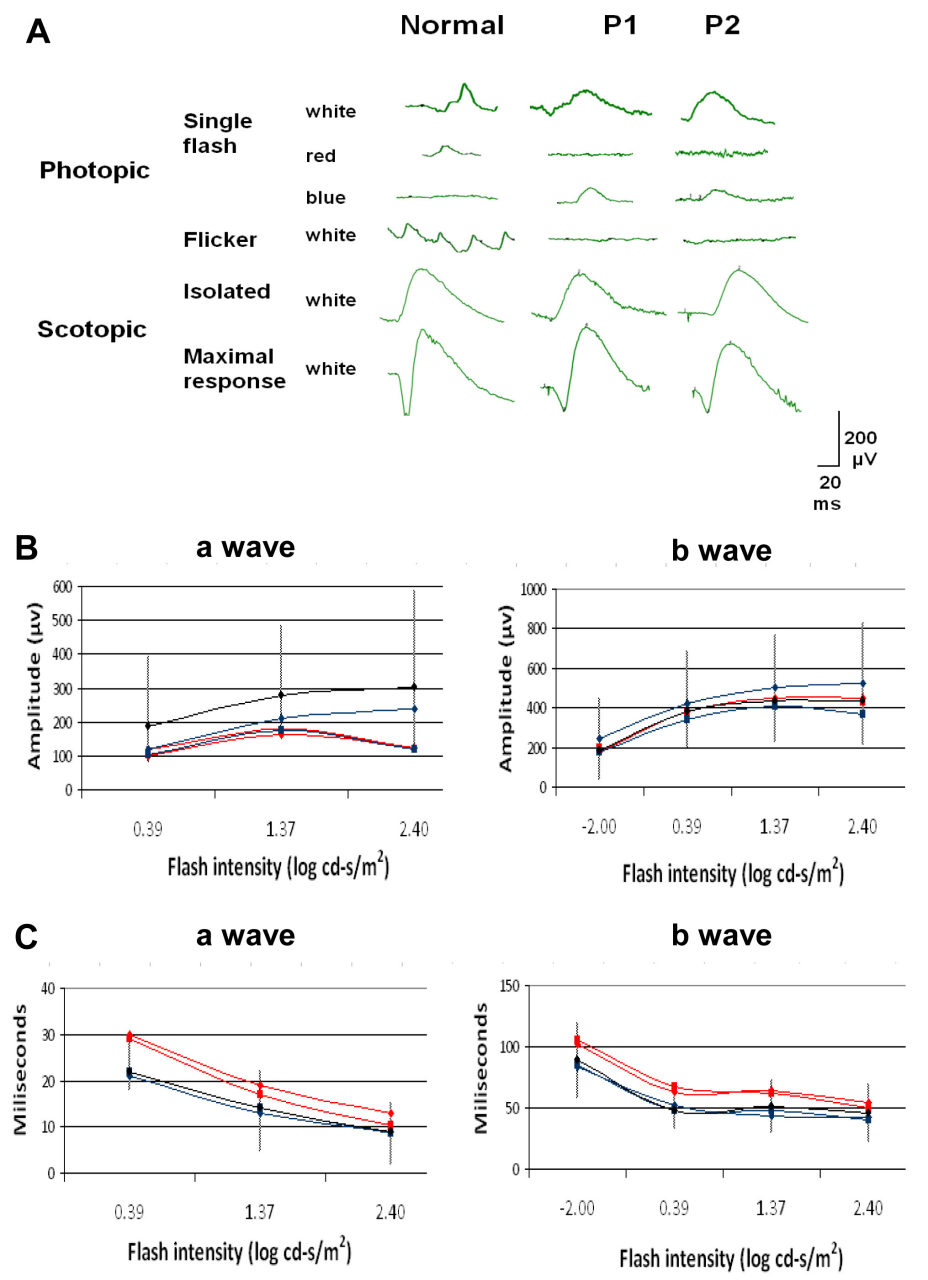
Electroretinograms from patient 1 (P1) and patient 2 (P2) compared to a normal subject. **A**: Photopic responses. No responses to red stimuli can be detected, whereas white stimuli show responses that are mostly within normal limits. Amplitudes of the response to blue stimuli are markedly higher than in normal subjects. Photopic 30-Hz flicker ERG responses are absent. Isolated rod b-wave amplitudes and maximal responses are generally within the normal limits (see **B**). **B**: Plots of scotopic response intensities from both patients. ERG intensities are shown as logarithmic values of stimulus intensity. P1 and P2 are represented in red and blue lines, respectively (one line for each eye). Black and Vertical lines represent the normal average and range from 100 normal eyes, respectively. As this parameter does not follow a normal (Gaussian) distribution but is skewed [15,16], to obtain the normal range the values were first converted to their log_10_ values, which normalizes the distribution. Mean values±2.5 standard deviations (SD) were then calculated and the values were converted back to their antilog values. The a-wave amplitudes (left) are generally within the lower normal limit (except for P1 at the lowest ERG intensity, where it is lower than normal). The b-wave amplitudes (right) are within normal limits at all light intensities tested. **C**: Latency plots of the scotopic responses from both patients. For normal values, the normal range was calculated as the mean±2.5 SD. The a-wave latencies (left) are within normal limits (except for P1 at the lowest ERG intensity, where it is longer than normal). The b-wave latencies (right) are within the normal limits at all intensities, other than in P1 at 2.44 cd-s/m^2^ (log=0.39).

Full-field scotopic ERG responses showed a- and b-wave amplitudes and latencies within the normal range (assessments of amplitude and latency for both a- and b-waves were based on ERG responses from 100 normal eyes). The a-wave amplitude was smaller in P1, but was within the lower limit of normal. The a-wave and the b-wave latencies were longer for both waves but within the upper normal limit (Figure 2). Photopic 30-Hz flicker ERG responses were absent. Photopic spectral ERG responses evoked by a blue (440–480 nm 50% pass) stimulus (2.44 cd-s/m^2^) revealed b-wave amplitudes of 97 µV and 63 µV for the right and left eye, respectively, In P1 and of 62 µV and 67 µV, respectively, in P2 (compared to 11±3 µV in the control). In contrast, b-wave amplitudes could not be detected in spectral ERG responses evoked by a red (510–580 nm 50% pass) stimulus (2.44 cd-s/m^2^). Response amplitudes and implicit times measured for the white stimuli were similar to those obtained for the responses to blue stimuli (Figure 2).

Mutation analysis of P1 was performed by direct sequencing of the following PCR-amplified exons: *NR2E3* (exons 1–8), *NRL* (exons 1–3), *THRB* (retinal-specific transcripts: exons 1 and 4–11), and *NR1D1* (exons 1–8). No potentially pathogenic mutations were detected in the coding region or in the exon/intron boundaries of these genes; however, the possibility of a mutation in the non-coding regions cannot be ruled out. The following nonpathogenic changes were identified: in *NR1D1*: the known homozygous single nucleotide polymorphism IVS5+40G>T (rs72836608) and a novel sequence change, IVS5–12_13insA; in *THRB*, a silent heterozygous sequence change was observed at rs3752874.

## Discussion

Based on the spectral photopic ERG responses observed in two Arab siblings with parental consanguinity, we diagnosed enhanced S-cone activity with higher than normal S-cone response and undetectable L-cone response. The photopic ERG responses seen in our patients resembled those seen in ESCS. However, unlike ESCS patients, who show marked peripheral pigment clumping, our patients had none. Moreover, the severely reduced scotopic ERG responses typical of ESCS patients [3,4] were not seen in our patients: scotopic b-wave amplitudes in both brothers were within normal limits and scotopic a-wave amplitude responses were found to be within the lower limit of normal with latencies within the upper normal limit. The somewhat borderline scotopic a-wave findings in our patients can be attributed to a slow a-wave composed of rods and S-cones without L- or M-cones. Moreover, it could be postulated that generation of the consecutive positive component of the ERG (the b-wave) prevented continuation of the relatively slower a-wave, resulting in a smaller a-wave. Further support for a new entity comes from the absence of peripheral retinal pathology and remarkable differences between the photopic and scotopic responses to high-intensity stimulus (unlike patients with ESCS, who show nearly identical photopic and scotopic responses [4]). Last, the negative results obtained on screening of candidate genes, including *NR2E3*, for pathological mutations also support a new entity.

The phenotype described in this study appears to reflect a new ocular genetic disease which, to the best of our knowledge, has never before been documented. Wright et al. [11] described one patient (“Patient A”) who was diagnosed with ESCS and exhibited some features resembling the phenotype that we describe here. However, whereas rod function in our patients was within the normal range, “Patient A” showed a unique clinical feature of moderate rod function in addition to pigment clumps at the retinal vascular arcades, which were not seen in our patients. “Patient A” also had no detectable mutation in the *NR2E3* gene, and it was suggested that the cause of his disease might be a digenic mechanism with a heterozygous *NRL* mutation and a mutation in another, unknown gene.

We attempted to identify the underlying molecular defect in our patients by analyzing the *NR2E3* gene, mutation of which is the classical cause of ESCS. No pathogenic mutation was detected. Bearing in mind the abovementioned suggestion of a heterozygous mutation with a digenic mechanism [11] we also analyzed *NRL*, but again found no pathogenic mutations. NRL forms part of a transcription regulatory complex with NR1D1 in vivo and synergistically activates the expression of rhodopsin. Two nonpathogenic homozygous changes were identified in *NR1D1*, but no pathogenic mutations were identified. Another related gene, *THRB*, was also excluded as the cause of disease in this family.

In their attempt to understand the molecular genetics of photoreceptor development, Bumsted O’Brien et al. showed that loss of *NR2E3* function in *rd7* mice was associated with an increased number of S-cones, suggesting that NR2E3 may function as a suppressor of S-cone differentiation [12]. Another transcription factor essential for photoreceptor differentiation is the *NRL* gene, which is expressed in rods but not in cones [2]. In humans, missense mutations in *NRL* are associated with autosomal dominant retinitis pigmentosa [13]. In mice, targeted deletion of *NRL* (*NRL*^−/−^) results in a complete loss of rods and a marked increase in S-cones, demonstrating a phenotype similar to that in human ESCS and mouse *rd7*. In these mice, no rod function or rod-specific gene is expressed (even during early retinal development), and no *NR2E3* transcripts are found, suggesting that NR2E3 is downstream of NRL in the transcription hierarchy [14].

It is possible that in our analysis of the *NR2E3* gene a mutation in this gene, although looked for, was missed. However, the unique phenotype seen in the two brothers implies the presence of another mutation, namely one that results in enhanced S-cone function together with normal rod function. One possibility is that another protein, as yet unknown, imposes negative inhibition, perhaps in a step upstream of NR2E3, on the embryonic development of rod and S-cones. This finding suggests that *NR2E3* mutations prohibit the development of photoreceptor precursors to the rod and L- and M-cones, causing ESCS in upstream embryogenesis. The S-cone normally develops from a pre-precursor that develops in turn into the former photoreceptor precursor and S-cones. It is possible that our two patients harbor a new mutation that causes the photoreceptor precursor to differentiate into rods and not L- and M-cones. The NRL protein, which was found to be located upstream of the NR2E3 protein [14], is probably not far enough upstream to cause this new phenotype. Further investigation will be needed to identify the specific mutation causing this phenotype.

In summary, we present evidence that a genetic defect in a gene other than *NRL* or *NR2E2* causes a new autosomal recessive clinical phenotype of enhanced S-cone activity with normal b-wave amplitude rod function. Identification of this mutation is a prerequisite for understanding the factors regulating photoreceptor development. This in turn is essential for gaining a better understanding of photoreceptor development when looking for factors promoting stem cells for retinal therapy in the future.
